# The serum biomarkers NSE and S100B predict intracranial complications and in-hospital survival in patients undergoing veno-venous ECMO

**DOI:** 10.1038/s41598-024-82898-3

**Published:** 2024-12-18

**Authors:** Janine Walther, Mathias Schmandt, Stefan Muenster, Stefan Franz X. Kreyer, Marcus Thudium, Felix Lehmann, Julian Zimmermann, Christian Putensen, Jens-Christian Schewe, Johannes Weller, Stefan Felix Ehrentraut

**Affiliations:** 1 Department of Anesthesiology and Intensive Care Medicine, University Hospital Bonn, Venusberg-Campus 1, 53127 Bonn, Germany; 2 Department of Neurology, University Hospital Bonn, Bonn, Germany; 3 Department of Anesthesiology and Intensive Care Medicine, University Hospital Rostock, Rostock, Germany

**Keywords:** Cell death in the nervous system, Diseases of the nervous system, Respiratory distress syndrome, Risk factors

## Abstract

Neurological complications in patients undergoing veno-venous extracorporeal membrane oxygenation (V-V ECMO) are challenging, with new intracranial pathologies posing a grave risk. We aimed to evaluate the utility of neuron-specific enolase (NSE) and S100B biomarkers for predicting outcomes in new-onset intracranial pathology during V-V ECMO. A retrospective analysis spanning 2013–2021 at a German university hospital was conducted. Cases with electronically available data on NSE and S100B serum levels, new intracranial pathologies (intracerebral hemorrhage [ICH], subarachnoid hemorrhage [SAH], cerebral ischemia, hypoxic-ischemic encephalopathy [HIE]), and survival during or after V-V ECMO were screened. The primary objective was to assess the prognostic value of NSE and S100B for in-hospital survival during V-V ECMO. Secondary objectives included analyzing clinical characteristics, outcome parameters, and biomarker distribution in V-V ECMO patients. Additionally, the prognostic value of NSE and S100B for in-hospital death and occurrence of intracranial pathology was calculated. Among 744 ECMO recipients, 426 underwent V-V ECMO. No significant differences in disease severity or organ failure scores were observed between groups, except for SAPS at discharge, which was higher in patients with new intracranial pathologies. Patients with new intracranial pathologies had lower median survival and higher in-hospital mortality. Weaning success from ECMO was also significantly reduced in these patients. Cut-off values of 58.4 µg/lfor NSE and 1.52 µg/l for S100B were associated with detrimental outcomes, characterized by significantly reduced median survival. A significant difference in maximum serum NSE concentration was found between patients with and without new intracranial pathology. All screened cases with new intracranial pathology had an unfavorable neurological outcome (modified Rankin Score [mRS] > 3) at discharge, with a higher proportion having an mRS of 6 in the high NSE group. The emergence of intracranial pathology during V-V ECMO significantly increases the risk of death. Changes in NSE and S100B levels serve as valuable follow-up parameters for predicting new intracranial pathology and survival during V-V ECMO therapy.

**Supplementary Information:**

The online version contains supplementary material available at 10.1038/s41598-024-82898-3.

## Background

Extracorporeal membrane oxygenation (ECMO) is a well-established salvage therapy for acute but potentially reversible respiratory failure in the treatment of acute respiratory distress syndrome (ARDS). Despite its positive impact on survival and physical functioning at 6 months^1^, mortality remains high and half of the patients receiving ECMO die from ARDS sequelae or ECMO complications^2^.

Central nervous system (CNS) complications during ECMO treatment represent a clinical challenge. CNS complications such as hypoxic-ischemic encephalopathy, intracranial hemorrhage or ischemic stroke occur in 5–13% of patients and are associated with increased mortality and worse clinical outcomes^3–5^. ECMO-specific factors including the need for systemic anticoagulation, damage to the blood compartment caused by physical shear stress, hemolysis, microinflammation, and endothelial cell damage during ECMO therapy, contribute to an elevated risk of intracranial hemorrhage^6^. Of note, the requirement for sedation of ECMO patients renders clinical neuromonitoring difficult and symptoms of CNS complications might only be noticed with significant delay upon reduction of sedation. Further, cranial MRI imaging during ECMO treatment is not available and computed tomography (CT) is associated with significant risks and logistical challenges due to the necessary transfer of ECMO patients. Taken together, these diagnostic challenges might lead to an underestimation of new intracranial pathologies during ECMO treatment^7^.

Accordingly, there is a high clinical need for biomarkers predicting CNS complications and neurological outcomes in ECMO patients. Candidate serum biomarkers are the neuron-specific enolase (NSE, soluble protein 14-3-2) known for its reliability in indicating axonal damage and subsequent regeneration^8^, and S100B, a subset of a family of calcium-binding proteins highly abundant within the nervous system, serving as a predictive biomarker especially in the context of inflammatory or degenerative processes^9^. In patients with veno-arterial ECMO (V-A ECMO) after cardiopulmonary resuscitation, both NSE and S100B levels were predictive for neurological outcomes following hypoxic-ischemic encephalopathy^10^. However, in veno-venous ECMO (V-V ECMO), these biomarkers and their impact on neurological outcomes and the presence of CNS complications is unclear due to the much more variable course of disease as opposed to V-A ECMO following an episode of cardiopulmonary resuscitation, and has not been sufficiently evaluated so far.

Leveraging a large retrospective cohort, we evaluate NSE and S100B as prognostic biomarkers in V-V ECMO with regard to CNS complications and neurological outcome.

## Materials and methods

### Study design and study participants

We performed a retrospective cohort analysis of all ECMO procedures at the author’s institution, a quaternary university hospital in Germany, between 01/2013 and 12/2021. Inclusion criteria include the following: age ≥ 18 years, V-V ECMO support, electronic medical records available, including ECMO and vital parameters as well as laboratory measurements. Patients with V-A ECMO or pumpless extracorporeal lung assist (pECLA) were excluded.

### Aim

Primary objective of the study is to evaluate the prognostic relevance of the serum biomarkers NSE and S100B for in-hospital survival in patients with V-V ECMO support.

Secondary objectives are:

- to describe the distribution of serum biomarkers in V-V ECMO patients.
- to investigate clinical characteristics of the cohort such as time on mechanical ventilation, weaning from ECMO support, and organ dysfunction (e.g. SOFA score)^11^.
- to measure outcome parameters such as survival times, in-hospital death, long-term survival, and neurological outcome.
- to calculate the prognostic value of NSE and S100B for in-hospital death and the occurrence of new intracranial pathologies.

### Indication for ECMO

Indication for V-V ECMO support was in compliance with the ELSO General Guidelines^12^. Indications included treatment of severe hypoxaemia, hypercapnia and prevention of harmful mechanical ventilation (i.e. prolonged use of exceedingly high peak inspiratory pressures or driving pressure > 15 cmH_2_0) to ensure sufficient gas exchange. All decisions for implantation were made following consensus between at least two experienced members of our ECMO team^13^.

### Indication for obtaining serum markers and indication to obtain neuroimaging

Serum biomarkers NSE and s100B are obtained daily as standard of care for all patients undergoing ECMO at our center, irrespective of clinical suspicion of neurologic injury. This is standard procedure to have additional indicators of neurologic pathologies unobtainable through clinical evaluation due to sedated state of patients.

If the patients’ clinical condition allows, neuroimaging is performed upon admission (for cases admitted via primary, i.e. out-of-hospital, ECMO implantation by our ECMO transport team^13,14^, if no previous cCT exists, or if clinical observations lead to suspicion of neurological injury.

### Disease severity

Disease severity was assessed using the “Sequential Organ Failure Assessment” (SOFA, GCS was scored as best assumed or last known value)^15^, “Simplified Acute Physiology Score II” (SAPS II)^16^, “Therapeutic Intervention Scoring System” (TISS-10)^17^and the “Charlson Comorbidity index” (CCI)^18^, using all available data at the time of scoring. Estimated survival and risk stratification for V-V ECMO was determined using the “Respiratory Extracorporeal Membrane Oxygenation Survival Prediction” (RESP) Score^19^. If any indication of a cardiopulmonary resuscitation (CPR) event preceding the start of ECMO was present, patients were classified as having undergone CPR. To specify the nature of the event, time till “Return of Spontaneous Circulation” (ROSC) was assessed and patients categorized into the following groups: “no CPR”, “in hospital cardiac arrest (IHCA) < 5min”, “IHCA, >5min”, “IHCA, unknown duration”, “out-of-hospital cardiac arrest (OHCA) < 5min, “OHCA, >5min” and “OHCA, unknown duration”. “no proven CPR” was recorded, if after reassessment of all available records, no CPR event was proven. We chose five minutes of CPR duration as discriminator, since favourable outcome is likely to be lowered if 5 minutes CPR time are exceeded^20^.

### Data acquisition

Outcomes recorded include survival and neurological functioning according to the modified Rankin Scale (mRS), where mRS 0–3 was defined as a favourable neurological outcome^21^. For the total survival time, an active follow-up for each patient was performed and data was censored at the last follow-up.

All cranial CT and MRI scans performed during the patients’ respective hospital episode were analyzed. The occurrence of new intracranial pathologies, comprising intracranial hemorrhages (both intracerebral hemorrhage (ICH) and subarachnoidal hemorrhage (SAH)), intracranial ischemia or signs of hypoxic-ischemic encephalopathy were assessed. The clinical relevance of the imaging findings was independently evaluated by two experienced physicians (neurologist (Ja.W.) and intensive care physician (M.S. or S.F.E.)). Findings were regarded as relevant if size/volume, location or the pathology itself (e.g. global hypoxia) had high likelihood of functional impact. Intracranial mass hemorrhage with midline shift and/or cerebral herniation or severe global hypoxia were always regarded as clinically relevant. Small/patchy diffusion restrictions, minor or small/atypical cortical SAH or age associated microangiopathies were considered considered clinically irrelevant.

Preexisting imaging findings, defined as known intracranial pathologies at the time of hospital admission, were not considered as new intracranial pathologies. The time point of cranial imaging was evaluated as sequential day following ECMO therapy initiation.

### Ethical approval/informed consent

Ethical approval for our study was provided by the Ethic Committee (No. 492/20) of the University Hospital Bonn, Germany and the need for informed consent was waived. Research was performed in accordance with the Declaration of Helsinki.

## Statistical analyses

All data are presented as median and interquartile range (IQR) for non-normally distributed or mean ± standard deviation (SD) for normally distributed continuous variables as appropriate, and as frequency distributions with percentages for categorical variables. The t-test was used to test group differences for norm-distributed variables, for non-normally distributed variables the Wilcoxon test was used. Categorial variables were assessed using Pearson’s Chi^2^- or Fisher’s Exact-test.

All tests were two-sided and *p* < 0.05 was preset as the cutoff for statistical significance. Due to the exploratory nature of the analysis, no adjustment for multiple testing was performed.

Laboratory values for each patient were aggregated and the maximum value during the hospital episode was recorded. Consecutive laboratory values were evaluated in reference to ECMO initiation (day 0).

To group patients into high or low serum marker groups, optimal cutoff values for the prediction of in-hospital survival were calculated using the “survminer” package (V.0.4.9) for R. Survival analysis was performed using the Kaplan-Meier survival estimate and the stratified log-rank test (LRT)^22^.

To determine hazard ratios (HRs) with 95% confidence intervals (CI) for NSE and S100B, a Cox proportional hazard model was used^23,24^. Marginal effect size for each serum marker in regard to survival as outcome parameter were calculated and a restricted cubic spline model was generated^25,26^. All analyses were performed in R version 4.1.2^27^.

## Results

### Cohort description

From 01/2013 to 12/2021, a total of 744 ECMO treatments were performed at our center. After exclusion of V-A ECMOs and pECLAs, 426 V-V ECMO runs were included in this study. The cohort consisted of 136 (32%) female and 290 (68%) male patients. Median age was 55.6 years (IQR 47;64). Further cohort characteristics are provided in Table 1.

**Table 1:** Cohort characteristics.

Variable	Metric	Value
Sex, n(%)	Female	136 (32%)
Age [years]	Median[IQR]	55.6 [47.0;64.0]
Height [cm]	Median[IQR]	175.0 [168.0;180.0]
Weight [kg]	Median[IQR]	90.0 [80.0;109.5]
BMI	Median[IQR]	29.2 [26.1;34.7]
Primary cause of ARDS, n(%)	unknown	1 (0.2%)
	Aspiration pneumonitis	36 (8%)
	Asthma	4 (1%)
	Bacterial pneumonia	68 (16%)
	Non-respiratory and chronic respiratory diagnoses	24 (6%)
	Other acute respiratory diagnosis	119 (28%)
	Trauma/burn	7 (2%)
	Viral Pneumonia	167 (39%)
	Total	426 (100%)
CCI	Median[IQR]	1.0 [0;2.0]
SOFA score at day 0 of ECMO	Median[IQR]	9.0 [7.0;10.0]
RESP score risk class	indeterminable	11 (2.5%)
	I	10 (2%)
	II	73 (17%)
	III	181 (42%)
	IV	120 (28%)
	V	31 (7%)
	Total	426 (100%)
RESP score	Median[IQR]	0 [-3.0;2.0]
Renal failure prior to ECMO, n(%)	Yes	119 (28%)
CPR prior to ECMO, n(%)	Yes	43 (10%)
CPR specifics, n(% of all recorded CPRs)	No proven CPR	7(16%)
	IHCA, <5 min	20(46%)
	IHCA, >5 min	8(19%)
	OHCA, <5 min	1 (0%)
	IHCA, unknown duration	1 (0%)
	OHCA, >5 min	6 (14%)
	OHCA, unknown duration	0

The median total length of stay of the cohort was 27.8 days [15.0;53.0]. Median time on ECMO support was 12 days (IQR 7.9;19.8) and weaning from the ECMO circuit was successful in 217 (51%) cases. The median time of survival was 33 days (IQR 14;367) and in-hospital death occurred in 256 (60%) of included cases. Further outcome characteristics are provided inTable 2.

**Table 2:** Outcome.

Variable	Metric	Total
Length of stay (days)	Median[IQR]	27.8 [15.0;53.0]
ICU length of stay (days)	Median[IQR]	25.1 [13.8;47.6]
Death	Yes	283 (66%)
in-hospital death	Yes	256 (60%)
days of survival	Median[IQR]	33.0 [14.0;367.0]

### Biomarker analysis

A total amount of 1298 serum levels for NSE and 1112 for S100B were analyzed. The number of individual patients with available serum levels was 157 for NSE and S100B, with a median number of measurements per patient of 5 [IQR 2;14.8] and 11 [IQR 5;16], respectively. The median maximum serum level of NSE (NSE max) was 57.8 µg/l [IQR 40.2;93.1] and the median maximal serum value of S100B (S100B max) was 0.4 µg/l [IQR 0.2;1.0]. The optimal cut-off value for predicting in-hospital demise for both serum biomarkers was derived using the Youdens-J index. The calculated cut-off values were 58.4 µg/l for NSE and 1.52 µg/l for S100B. To rule out the possibility, that the seemingly high proportion of patients with CPR events preceeding ECMO skew the biomarker cut-off due to their higher likelihood of brain damage, we performed subgroup analyses. When all patients with CPR were excluded, the cut-off values for S100B remained unchanged, while the cut-off value for NSE minimally increased (61.8 µg/l vs. 58.4 µg/l). In the Kaplan-Meier Analysis, the p-value for log-rank test for trend remained significant (*p* = 0.036). The observed median maximal values for NSE and S100B did not differ significantly between patients with or without CPR (data not shown). When only patients with the highest likelihood for neurological damage, due to prolonged CPR > 5 min, were excluded, cut-off values were unchanged compared to the initial analysis.

### Survival based on serum biomarker levels

**Fig. 1:**
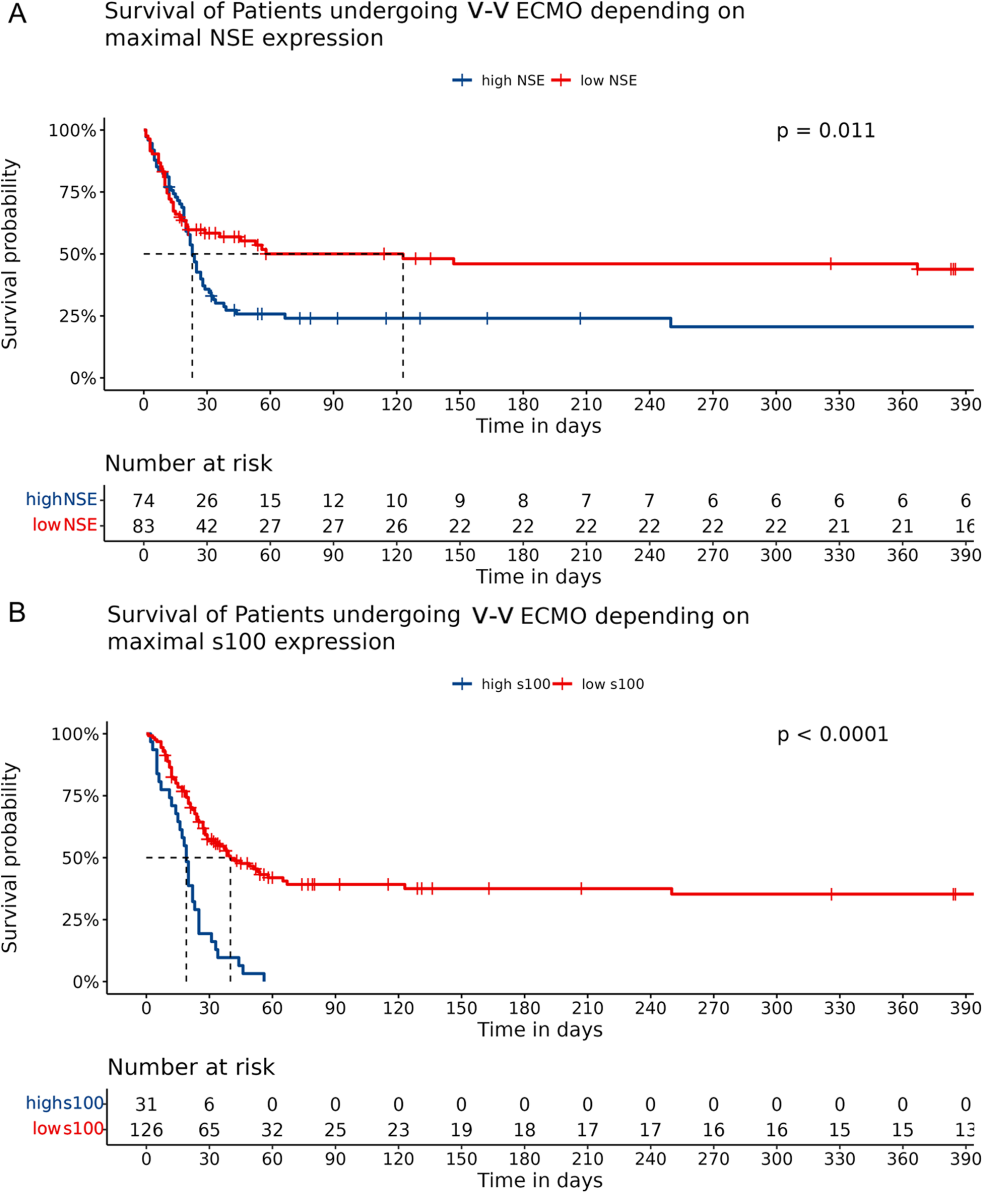
Survival curves and risk tables depicting long-term survival depending on maximal values of NSE (**A**) or S100B (**B**). low NSE: NSE below 58.4 µg/l, low S100B: below 1.52 µg/l. Median survival, i.e. the time point where 50% of patients were alive is indicated by dashed lines.

Survival times differed significantly between patients dichotomized according to the calculated cutoff-value for the respective serum marker. Patients with high NSE levels (i.e. observed maximal value above 58.4 µg/l) had significantly reduced median survival times (23 vs. 123 days, LRT *p* = 0.011, Fig. 1A) compared to those with low NSE levels. This was also true in patients showing a maximal S100B serum value above 1.52 µg/l, where median survival was significantly reduced (19 vs. 40 days, LRT *p* < 0.0001, Fig. 1B).

High serum biomarker levels were also associated with higher in-hospital and total mortality. For further detailed comparison of ICU outcomes according to dichotomized maximum NSE and S100B levels, please refer to Table 3.

**Table 3:** Serum biomarker group outcomes.

Variable	Metric	NSE group		*p*-value	S100B group		*p*-value
		Low NSE	High NSE		Low S100B	High s100B	
Length of stay	Median[IQR]	25.9 [12.5;47.5]	25.9 [15.3:42,7]	ns	29.7 [18.8; 52.9]	19.9 [13.6;28.4]	0.0017
ICU length of stay	Median[IQR]	21.6 [11.1;42.5]	25.9 [15.2;39.8]	ns	28.5 [18.4;50.8]	19.9 [13.6; 28.5]	0.0042
Death	Yes	45 (54%)	55 (74%)	0.009	70 (56%)	31 (100%)	< 0.001
In-hospital death	Yes	42 (51%)	56 (76%)	0.0012	71 (56%)	31 (100%)	< 0.001
Days of survival	Median[IQR]	31.0 [11.0;346.5]	23.0 [13.2; 42.0]	ns	32.0 [17.2: 59.5]	19.0 [11.5; 25.0]	0.0002

Univariable Cox regression confirmed a detrimental hazard ratio (HR) for in-hospital mortality in patients with high maximum biomarker levels for both NSE and S100B (high NSE: HR 1.6; CI 1.12–2.51; *p* = 0.0117; high S100B: HR 3.37; CI 2.18–5.24; *p* < 0.0001).

**Fig. 2:**
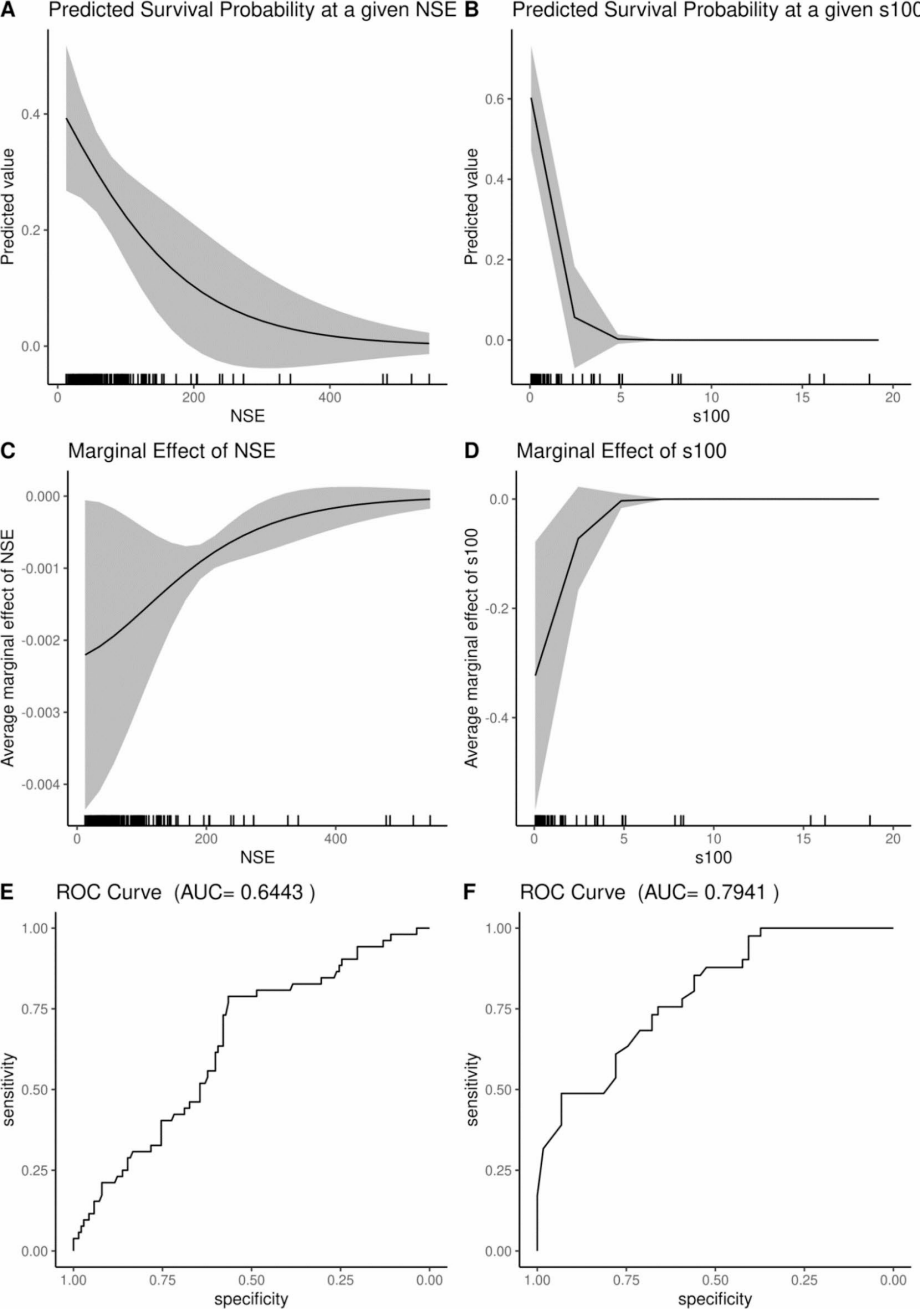
Univariable models predicting survival. A/B: predicted survival for any given biomarker value (**A**: NSE, **B**: S100B). (**C**/**D**): marginal effect size illustrating the impact of biomarker changes on survival at the respective biomarker values (**C**: NSE, **D**: S100B). (**E**/**F**): Receiver Operating Curves for each model (E: NSE, F: S100B).

The predicted survival probabilities, derived from logistic regression analysis including the respective biomarkers as continuous predictors, decrease rapidly for values up to 200 µg/l (NSE) and 2.8 µg/l (S100B), respectively (Fig. 2A and B). Higher biomarker levels were already associated with a low survival probability and further increase had less additional impact on survival. Accordingly, the size of the average marginal effect for each biomarker decreases above an NSE of 200 µg/l and S100B of 2.8 µg/l as depicted in Fig. 2C and D. Receiver Operating Characteristic (ROC) curves for the corresponding univariable logistic regression models show a higher area under the curve (AUC) for the model based on S100B (AUC: 0.79) compared to the univariable model based on NSE (AUC: 0.64, Fig. 2E and F).

### New intracranial pathologies

309 CT and 12 MRI scans in 121 patients were evaluated for the occurrence of new intracranial pathologies. 88 patients (21%) developed a new intracranial pathology during V-V ECMO treatment. CT scans showed 153 newly detected intracranial pathologies (ischemic stroke *n* = 35, SAH *n* = 37, ICH *n* = 56, hypoxic-ischemic encephalopathy *n* = 25). Of those, 83 were regarded as clinically relevant (ischemias *n* = 14, ICH *n* = 28, SAH *n* = 18, hypoxic-ischemic encephalopathy *n* = 23). The 12 MRI scans showed four new and clinically relevant pathologies (2 ischemic strokes, 2 cases with hypoxic-ischemic enephalopathy).

There were no differences in demographical variables between patients with and without newly diagnosed intracranial pathology (for a detailed cohort description, including prior medical history indicated by the Charlson Comorbidity index, the primary cause of ARDS and preexisiting organ failure, consult Table 1). Disease severity and organ failure scores (SOFA, SAPS, TISS, RESP score, CCI) at admission, 24 h after ECMO initiation and at ICU discharge were assessed and compared between patients with vs. without new intracranial pathologies. Here, only SAPS at discharge differed between groups, with patients with new intracranial pathologies showing higher scores (median 55.5 [IQR 41.2:61:8 vs. no intracranial pathology: median 44 [IQR 31:59], *p* = 0.007, for detailed comparison refer to Table 4.

**Table 4:** Disease parameters and outcomes by new headpathology.

variable	metric	New intracranial pathology		*p* value
		No	yes	
SAPS 24 h after ECMO initiation	Median[IQR]	46.0 [38.0;55.0]	44.0 [37.0;52.0]	ns
SAPS at discharge	Median[IQR]	44.0 [31.0;59.0]	55.5 [41.2;61.8]	0.0069
TISS 24 h after ECMO initiation	Median[IQR]	27.0 [23.0;33.0]	28.0 [23.0;34.0]	ns
TISS at discharge	Median[IQR]	22.0 [10.0;30.0]	29.0 [22.2;33.0]	0.0003

**Fig. 3:**
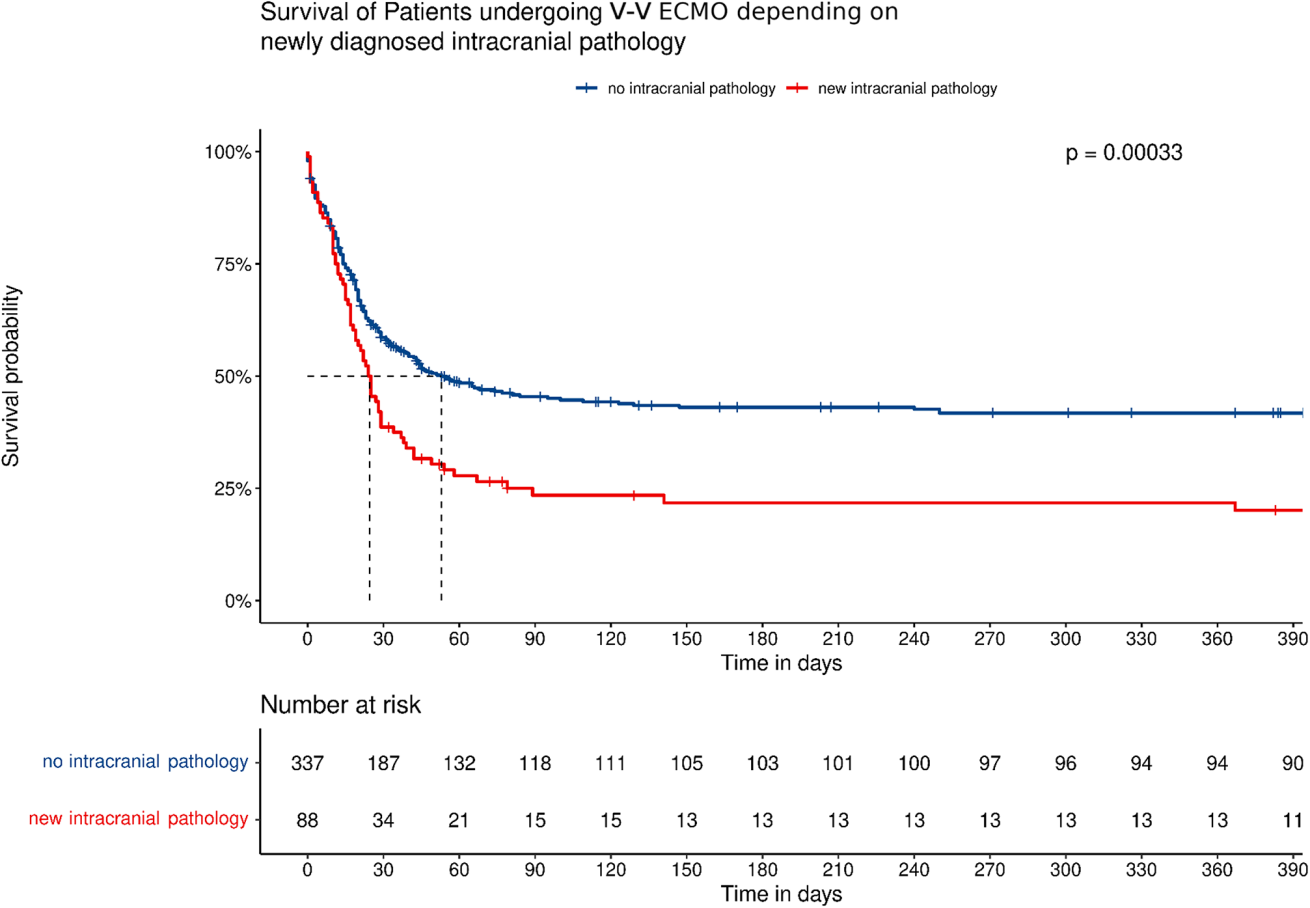
Kaplan-Meier analysis and risk table of patients with (red) and without (blue) newly diagnosed intracranial pathologies. If a new intracranial pathology is observed during ECMO support, median survival time is significantly reduced (log-rank test for trend, *p* = 0.00033).

New intracranial pathologies were associated with a significantly reduced overall survival: the median survival was 24.5 days for patient with a new intracranial pathology and 53 days for patients without it (LRT *p* = 0.0003, Fig. 3). The association of new intracranial pathologies with reduced in-hospital survival was confirmed in Cox regression models (HR 1.66; CI 1.25–2.19; *p* < 0.0005). The individual hazard ratios for in-hospital death for specific intracranial pathologies are listed in Table 5.

**Table 5:** Hazard ratios (HR) for specific intracranial pathologies and their impact on the outcome variable in-hospital death.

Variable	HR	CI [5–95%]	*p*-value
SAH	2.1	1.44–3.06	< 0.001
ICH	1.6	1.09–2.27	0.015
Ischemic stroke	1.22	0.76–1.94	ns
Hypoxic-ischemic encepalopathy	3.78	2.07–6.97	< 0.001

**Fig. 4:**
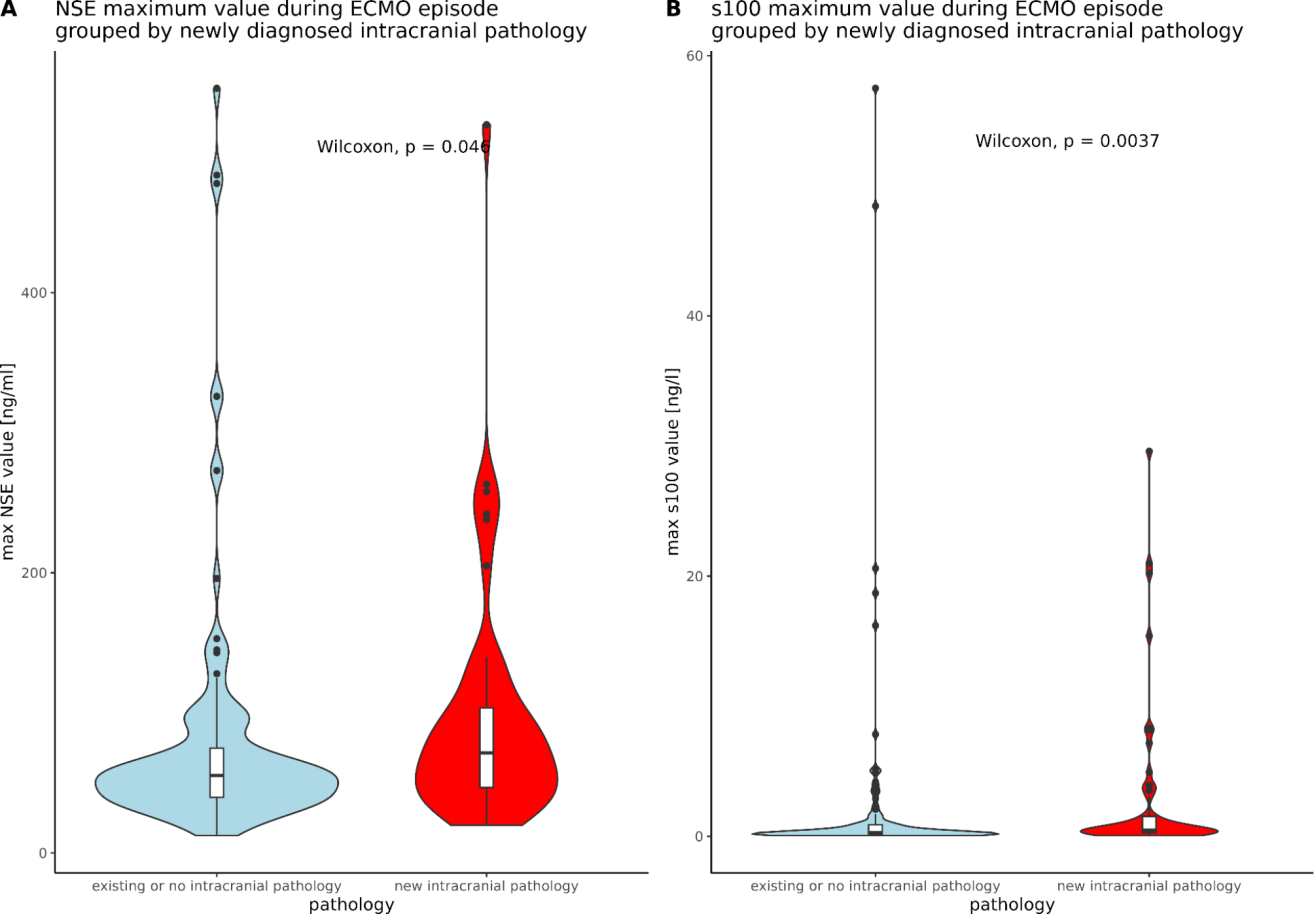
Serum biomarker levels differed significantly between patients with vs. without a newly diagnosed intracranial pathology during ECMO treatment. (**A**): NSE levels of patients with new intracranial pathology vs. patients without intracranial pathology (*p* = 0.046) and (**B**): S100B levels of patients with new intracranial pathology vs. patients without intracranial pathology (*p* = 0.0037).

As expected, maximal observed NSE levels were significantly higher in patients with new intracranial pathologies compared to non-affected patients (71.4 µg/l [IQR 46.7;103.5] vs. 55.2 µg/l [IQR 39.7;74.8], *p* = 0.046, Fig. 4A). The same was observed for S100B, where serum levels were higher in patients with a newly detected intracranial pathology (0.5 µg/l [IQR 0.3;1.5] vs. 0.3 µg/l [IQR 0.1;0.9], *p* = 0.0037, Fig. 4B).

ECMO weaning success was significantly reduced in patients with intracranial pathologies (33% vs. 56%, *p* < 0.001, Pearson’s Chi^2^-test, reasons for ECMO weaning failure, i.e. the therapy refractory condition leading to death is supplied as supplemental Table 1). While the time spent in hospital and on ICU did not differ between patients who suffered from new intracranial pathologies, both overall and in-hospital death occurred significantly more often in the group with newly diagnosed intracranial pathologies Table 6.

**Table 6:** Outcomes respective of new intracranial pathology.

Label	Metric	New intracranial pathology		*p*-value
		No	Yes	
Length of stay	Median[IQR]	28.6 [15.8;53.9]	23.9 [14.3;42.6]	ns
ICU length of stay	Median[IQR]	26.7 [13.9;48.5]	23.1 [13.5;41.7]	ns
Death	Yes	213 (63%)	70 (80%)	0.0034
in-hospital death	Yes	188 (56%)	68 (77%)	0.0002
days of survival	Median[IQR]	38.0 [14.0;432.0]	24.5 [11.8;55.0]	0.0136

### Reasons for death or withdrawal of therapy

As described above, 256 patients died during the hospital episode in which the ECMO treatment occurred. The reasons for death included therapy refractory disease, e.g. continued pulmonary failure or multi-organ failure despite all therapeutic efforts, withdrawal due to neurological/neurosurgical conditions untreatable under ECMO or unfavourable neuroprognosis (e.g. intracranial mass bleeding, cerebral herniation, global hypoxic brain damage) or withdrawal based on existing living-will or decision to end therapy by legal representative / next-of-kin. No significant difference between patients with successful ECMO weaning and patients without successful ECMO weaning were observed in regard to reason for death Table 7.

**Table 7:** Reasons for in-hospital death.

		Successful ECMO Weaning	
		Yes	no
Withdrawal due to neurological/neurosurgical pathologies, n(%)	yes	3(7%)	19(9%)
	no	43(93%)	188(91%)
Death due to therapy refractory disease, n(%)	yes	39(85%)	182(88%)
	no	7(15%)	25(12%)
Withdrawal due to non neurological conditions, n(%)	yes	41(89%)	201(97%)
	no	5(11%)	6(3%)

Since this was a retrospective study, all radiography images and their respective grading in regard to clinical relevance were performed after the patients were treated, the findings, be it neuromarkers or radiology findings, were not part of the clinical decision process.

### Diagnostic yield of neuroimaging according to serum biomarker level changes

**Fig. 5:**
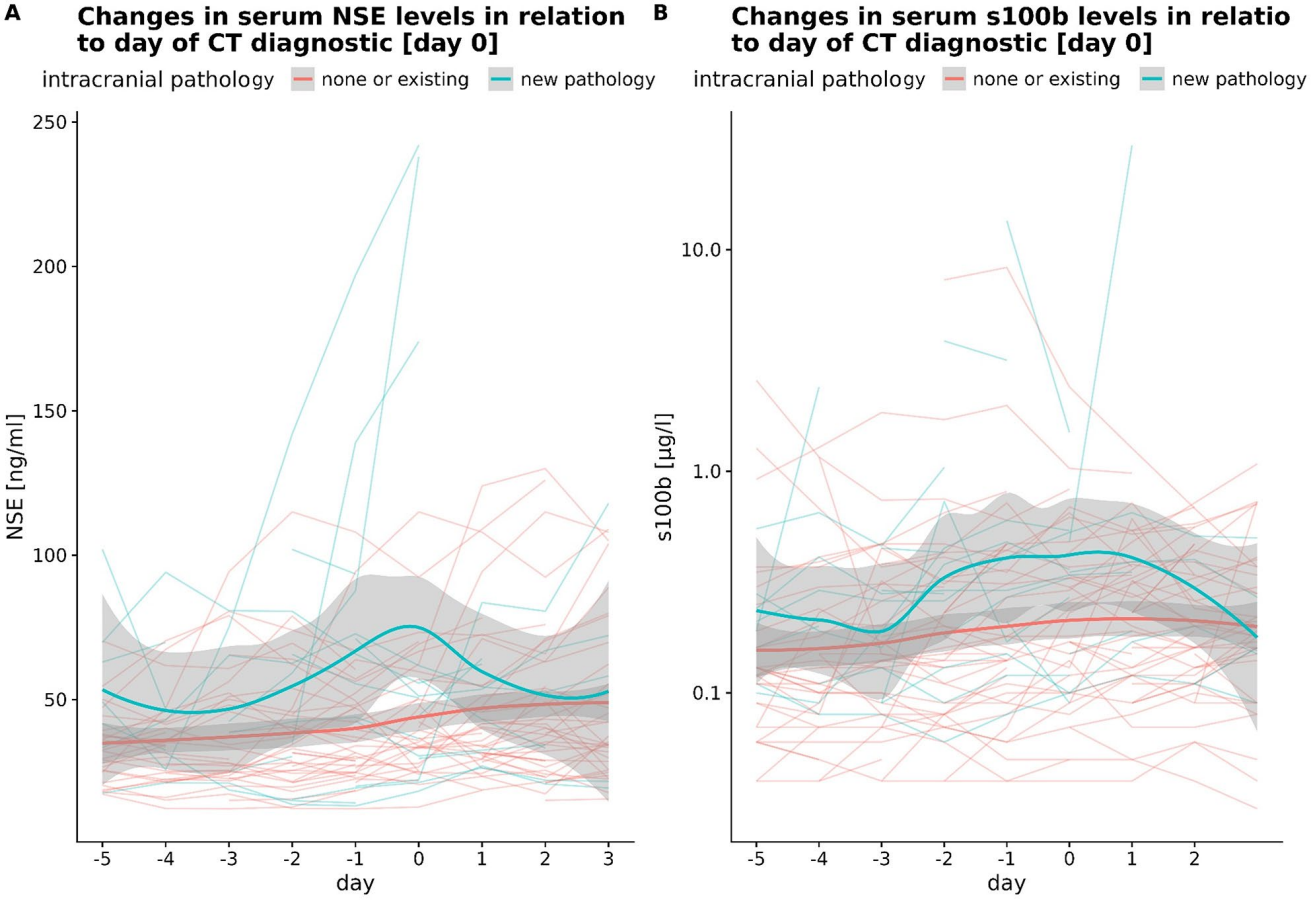
(**A**): A transient increase of patients’ NSE levels in regard to the date of cranial CT (day 0) is observable in patients with a new intracranial pathology. (**B**): Transient increase of S100B starting at day − 2 and remaining until the day after the diagnostic procedure visible in patients with newly observed intracranial pathology. Both panels: Coloured line represents the smoothed conditional means based on the individuals’ levels (faint lines) using a Loess fitting. Grey ribbon indicates standard error of predicted means.

We hypothesized that dynamic changes of the serum biomarkers NSE and S100B might precede the diagnosis of new intracranial pathologies. Indeed, both NSE and S100B show an increase around the time of radiological diagnosis of a new intracranial pathology (Fig. 5A). For NSE levels, this increase was most profound on the day before and the day of confirmative imaging. S100B showed a less pronounced and longer increase from 2 days before until the day after diagnostics (Fig. 5B). While an observed maximal S100B above 1.52 µg/l is predictive of poor outcome, values between 0.3 and 0.7 have a high likelihood of detecting a new pathology if timely neuroimaging is performed.

### Neurological outcomes

**Fig. 6:**
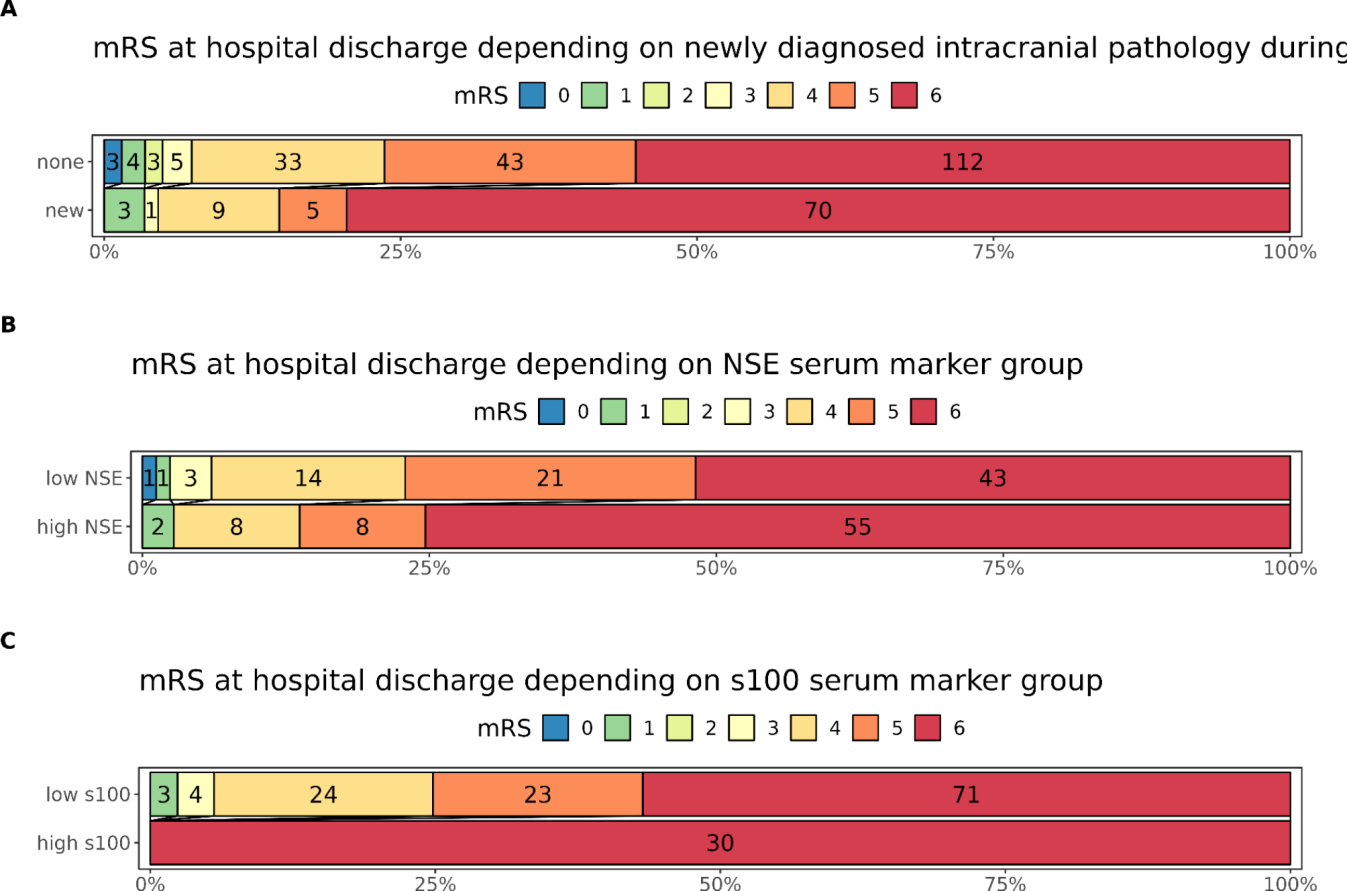
Distribution of modified Rankin Scale scores at hospital discharge; 0 indicates no symptoms, 1 indicates no clinically significant disability, 2 minor clinical disabilities, 3 indicates moderate disability but the ability to walk unassisted, 4 moderate to severe disability, 5 severe disabilities, 6 death. Number of patients in each bracket provided as absolute number. **A** New onset of intracranial pathology increases the proportion of patients with unfavorable outcomes (mRS > 3). **B-C** Shift towards unfavorable outcomes in patients with serum levels of NSE or S100B above the calculated cut-off-levels (high NSE: NSE maximum level > 58.4 µg/l, high S100B; S100B maximum level above 1.52 µg/l).

An unfavourable neurological outcome (mRS > 3) at discharge was observed in 278 (95%) of evaluable cases. In patients with a new intracranial pathology, an unfavourable outcome was significantly more frequent (83 vs. 76%, *p* = 0.001 Fisher’s Exact Test, Fig. 6A). Patients with a maximal NSE value as defined above experienced an unfavourable outcome in 97 vs. 94% (*p* = 0.012, Fig. 6B). Further, a significantly higher proportion of patients in the high NSE cohort had a mRS of 6 (i.e. death; 55/74 (75%) vs. 42/83 (51%), *p* = 0.01 Fisher’s Exact Test for count Data, Fig. 6B). All patients with a S100B value above 1.52 µg/l died (mRS 6 in 30/30 cases (100%)), while patients with a maximal S100B below the threshold value had mRS values > 3 in 94% (*p* = 0.0001, Fig. 6C).

### Multivariable model

Finally, we investigated the combined prognostic impact of intracranial pathologies and serum biomarker levels. Multivariable Cox regression analysis confirmed an increased HR for in-hospital death for both new intracranial pathologies and increased serum biomarkers. Of note, the impact of increased S100B was numerically larger as compared to NSE (Table 8). Multivariable logistic regression models including the respective biomarker levels as continuous variables in addition to new intracranial pathologies confirmed the prognostic impact of both S100B and new intracranial pathologies, while NSE was not prognostic in the combined model (Table 8).

**Table 8:** Hazard ratios (HR) and odds ratios (OR) for intracranial pathologies and increased serum biomarkers on the outcome variable in-hospital death.

Multivariable logistic regression	Variable	OR	95% CI	p-value
Model 1	Intracranial pathology	3.88	1.72–9.67	0.002
	NSE	1.00	1.00-1.01	0.169
Model 2	Intracranial pathology	3.78	1.61–9.73	0.003
	S100B	3.03	1.6–7.84	0.006

Multivariable Cox regression	Variable	HR	95% CI	*p*-value
Model 1	Intracranial pathology	1.67	1.10–2.54	0.015
	Max NSE > 58.4 µg/l	1.53	1.01–2.31	0.045
Model 2	Intracranial pathology	1.68	1.13–2.51	0.010
	Max S100B > 1.52 µg/l	3.25	2.09–5.06	< 0.001

## Discussion

This study aimed to evaluate the use of serum biomarkers NSE and S100B for prognostication and detection of CNS complications in V-V ECMO. To this end, we analyzed > 1000 serum levels of S100B and NSE in 426 V-V ECMO patients. Our findings show that patients with maximal serum markers above 58,4 µg/l NSE or 1.52 µg/l S100B had reduced survival and higher rates of poor neurological outcome (mRS > 3), and both elevated biomarker levels and newly diagnosed intracranial pathologies during V-V ECMO were independently associated with reduced survival.

Considering the severity of the disease and the enormous effort involved in treating patients on ECMO, an early and reliable prognostic assessment is essential for patients and their treating teams. Particularly in patients with concomitant ICH, there is an additional risk that an overly pessimistic assessment of the prognosis leads to early discontinuation of therapy, resulting in poor outcomes. This so-called self-fulfilling prophecy has already been well described in ICH patients without ECMO therapy^28^. Do-not-resuscitate orders are an independent predictor of poor outcomes in these patients^29^. Surprisingly, avoidance of early treatment limitations in ICH patients can lead to a significantly lower case fatality than predicted^30^. This fact shows how important it is to base the medical evaluation of these patients on as many factors as possible to provide patient and family with the best possible therapy/counseling.

However, timely detection of cerebral complications and prognostication poses significant challenges. Clinical neurological assessment is limited because deep sedation is usually required during ECMO support. Cerebral imaging has not been evaluated as a screening tool and is associated with significant logistical challenges in patients on ECMO support, although these might be attenuated by bedside imaging facilities including portable CT or MRI. Further, invasive neuromonitoring procedures such as intracranial pressure monitoring or cerebral microdialysis are not feasible during ECMO support due to the increased risk of bleeding complications. For this reason, non-invasive monitoring methods, such as biomarkers for detection of emerging intracranial pathologies, NIRS (cerebral near infrared spectroscopy), and transcranial doppler sonography (TCD), could be useful^31^.

S100B and NSE have been used as markers of brain damage in various diseases^32^. In the context of ECMO patients, the combination of NSE and S100B with clinical examination findings has been used to predict survival after resuscitation from cardiac arrest^33^. This suggests that NSE and S100B levels may have prognostic value in patients receiving ECMO. Data on neurological outcomes after V-A ECMO and cardiac arrest are available for pediatric patients. These findings suggest that worse outcomes depend on the duration of resuscitation prior to rescue ECMO^34^. Floerchinger et al. back these findings in adult patients after cardiac arrest, with significantly higher mortality and poor neurological outcome when NSE levels were elevated above 100 µg/l^35^. Schrage et al. observed similar detrimental outcomes at even lower NSE serum levels of 70 µg/l^36^. In a recent, albeit small patient cohort of V-V ECMO patients, Burzyńska et al. found NSE levels of > 28.9 µg/l to be associated with increased mortality^37^. Czimmek et al. recently observed that serum levels of cardiac arrest survivors above 60 µg/l were associated with poor neurological outcome^38^. The threshold of 1.52 µg/l for S100B identified in our study is higher than the 1 µg/l cutoff reported in previous research^39,40^. This discrepancy may result from differences in patient populations, assay methods, and timing of sample collection. Our higher threshold may offer greater specificity in predicting adverse outcomes within our cohort but may reduce sensitivity compared to lower thresholds. This suggests that while patients with S100B levels above 1.52 µg/l are at a significantly increased risk, those with levels between 1.0 and 1.52 µg/l should not be overlooked. Further studies are needed to standardize S100B measurement protocols and establish universally applicable cut-off values to enhance its prognostic utility in clinical practice.

We extend these findings now to the level of both survival and functional outcome parameters in patients undergoing V-V ECMO, as both survival and functional outcomes are significantly reduced if NSE and S100B are elevated above the determined cut-off values of 58.4 µg/l for NSE or 1.52 µg/l for S100B. To the best of our knowledge, this is the first study with a large patient cohort to show that these markers can provide valuable information about brain damage and neurological outcomes in patients on V-V ECMO.

Further prospective research, including studies designed to validate our model and incorporating corrections for multiple comparisons, will be necessary to fully assess the predictive value and clinical implications of these findings. This is especially important, since NSE and S100B do not have the same predictive value, when used in combination with newly occurring intracranial pathologies. Additional markers of CNS damage, including neurofilament light chain, tau protein, and glial fibrillary acidic protein, also warrant investigation as they might have an even higher sensitivity and specificity than NSE or S100B^41^. While NSE and S100B are increased in the setting of hemolysis^42,43^ - frequently occurring during ECMO treatment - these novel markers might overcome this limitation, but remain yet to be evaluated in ECMO patients not suffering from cardiac arrest. Of note, they are not yet available in a timely fashion for most centers, and thereby currently not available in clinical routine.

## Discussion of limitations

Several limitations of our single-center, retrospective study must be acknowledged. First, there is a potential for selection bias, if neuroimaging or serum marker collection was performed only in patients with suspected or proven neurological damage. However, we believe this bias is minimal, as sampling was not restricted to patients with suspected or proven intracranial pathology. Second, our multivariate analysis did not include post-hoc testing, which could increase the risk of false-positive results. Given the exploratory nature of the study, our focus was on identifying potential associations between serum markers and adverse outcomes, with the understanding that future research will need to validate these findings and incorporate corrections for multiple comparisons. Third, we did not control for hemolysis, which may have contributed to the observed increases in serum markers. Nevertheless, the strong association between elevated markers and in-hospital mortality remains clinically relevant, irrespective of the source of marker elevation. Additionally, we observed increased diagnostic yield, with more intracranial pathologies identified on CT scans in patients with elevated serum markers, further supporting their prognostic utility. Fourth, the threshold for S100B in our study (1.52 µg/l) was higher than that reported in previous literature. This may reflect differences in patient populations, assay methods, and sample timing, and while this higher threshold increases specificity, it may reduce sensitivity for identifying at-risk patients with lower S100B levels. Lastly, the potential influence of serum markers on therapy withdrawal decisions was mitigated by the retrospective nature of the analysis; serum markers did not influence clinical decisions. In the 22 cases where therapy was withdrawn, this was due to clear evidence of highly relevant intracranial pathologies, such as global hypoxia or massive intracranial hemorrhage. These limitations underscore the need for prospective studies to confirm our findings and address these gaps.

However, owing to the paucity of data regarding serum markers to predict neurological and overall outcomes, we believe that this study provides valuable insights: in addition to cerebral complications, both NSE and S100B elevations were independently associated with survival and functional outcomes in V-V ECMO patients. Also, in regard to the ongoing debate about the correct anticoagulation strategy, with a higher occurrence of ICH in patients receiving anticoagulatory therapy with a higher target PTT, a serum biomarker as screening tool for intracranial pathologies would be valuable. The observed biomarker increases preceding the diagnosis of intracranial pathologies renders biomarker-guided cerebral imaging a prudent approach for monitoring these critically ill patients and deserves further evaluation.

## Electronic supplementary material

Below is the link to the electronic supplementary material.

Supplementary Material 1

## Data Availability

Availability of data and materials: Data on which the conclusions are drawn are available upon reasonable request from the corresponding author.

## Abbreviations

ARDS: Acute Respiratory Distress Syndrome
AUC: Area under the curve
CCI: Charlson Comorbidity Index
CI: Confidence interval
CNS: Central nervous system
CT: Computer tomography
CPR: Cardiopulmonary resuscitation
ECMO: Extracorporeal membrane oxygenation
ELSO: Extracorporeal Life Support Organisation
GCS: Glasgow Coma Scale
HIE: Hypoxic ischemic encephalopathy
HR: Hazard ratio
ICH: Intracerebral hemorrhage
ICU: Intensive care unit
IHCA: In-hospital cardiac arrest
IQR: Interquartile range
LRT: Log-rank-rest
Max: Maximum
Min: Minimum
MRI: Magnetic resonance imaging
mRS: Modified Rankin Score
NIRS: Near infrared spectroscopy
NSE: Neuron-specific enolase
OHCA: Out-of hospital cardiac arrest
pECLA: Pumpless extracorporeal lung assist
PTT: Partial Thromboplastin Time
RESP: Respiratory Extracorporeal Membrane Oxygenation Survival Prediction
ROC: Receiver operating curve
ROSC: Return of Spontaneous Circulation
SAH: Subarachnoid hemorrhage
SAPS: Simplified Acute Physiology Score
SD: Standard deviation
SOFA: Sequential organ failure assessment
TCD: Transcranial doppler
TISS: Therapeutic Intervention Scoring System
V-A: Veno-arterial
V-V: Veno-venous
