# Supplementary material for: The serum biomarkers NSE and S100B predict intracranial complications and in-hospital survival in patients undergoing veno-venous ECMO
Source: Sci Rep. 2024 Dec 18;14:30545. doi: 10.1038/s41598-024-82898-3 (PMC11655984; doi:10.1038/s41598-024-82898-3)
Supplement: Supplementary file 1 — Supplementary Material 1 [file 41598_2024_82898_MOESM1_ESM.docx]

Suppl. Table 1

| cause of death for patients not weaned from ECMO | not retrievable from records | 5 (3%) |
| --- | --- | --- |
|  | hemorrhagic shock | 9 (5%) |
|  | intraabdominal bleeding | 2 (1%) |
|  | intrapulmonary bleeding | 7 (4%) |
|  | Liver failure | 10 (5%) |
|  | (multi-)organ failure | 55 (30%) |
|  | non-occlusive mesenteric ischemia | 3 (2%) |
|  | therapy refractory respiratory failure | 44 (24%) |
|  | Sepsis/septic schock multiorgan failure | 46 (25%) |
